# Projecting Drivers of Human Vulnerability under the Shared Socioeconomic Pathways

**DOI:** 10.3390/ijerph15030554

**Published:** 2018-03-19

**Authors:** Guillaume Rohat

**Affiliations:** 1 Institute for Environmental Sciences, University of Geneva, 1205 Geneva, Switzerland; guillaume.rohat@unige.ch; Tel.: +41-22-379-3365; 2 Faculty of Geo-Information Science and Earth Observation, University of Twente, 7522 NB Enschede, The Netherlands

**Keywords:** Shared Socioeconomic Pathways, climate change, vulnerability, projections, Europe

## Abstract

The Shared Socioeconomic Pathways (SSPs) are the new set of alternative futures of societal development that inform global and regional climate change research. They have the potential to foster the integration of socioeconomic scenarios within assessments of future climate-related health impacts. To date, such assessments have primarily superimposed climate scenarios on current socioeconomic conditions only. Until now, the few assessments of future health risks that employed the SSPs have focused on future human exposure—i.e., mainly future population patterns—, neglecting future human vulnerability. This paper first explores the research gaps—mainly linked to the paucity of available projections—that explain such a lack of consideration of human vulnerability under the SSPs. It then highlights the need for projections of socioeconomic variables covering the wide range of determinants of human vulnerability, available at relevant spatial and temporal scales, and accounting for local specificities through sectoral and regional extended versions of the global SSPs. Finally, this paper presents two innovative methods of obtaining and computing such socioeconomic projections under the SSPs—namely the scenario matching approach and an approach based on experts’ elicitation and correlation analyses—and applies them to the case of Europe. They offer a variety of possibilities for practical application, producing projections at sub-national level of various drivers of human vulnerability such as demographic and social characteristics, urbanization, state of the environment, infrastructure, health status, and living arrangements. Both the innovative approaches presented in this paper and existing methods—such as the spatial disaggregation of existing projections and the use of sectoral models—show great potential to enhance the availability of relevant projections of determinants of human vulnerability. Assessments of future climate-related health impacts should thus rely on these methods to account for future human vulnerability—under varying levels of socioeconomic development—and to explore its influence on future health risks under different degrees of climate change.

## 1. Introduction

It has long been acknowledged that socioeconomic determinants play an important role in the characterization of climate risks, through vulnerability and exposure [1]. As a result, nearly all assessments of climate risks consider both climatic (hazard) and socioeconomic (vulnerability and exposure) conditions [2]. Nevertheless, when it comes to modelling future climate-related health risks, the overwhelming majority of studies have been based on projections of future climatic conditions—through climate models and scenarios—superimposed on current socioeconomic conditions only [3,4,5]. By making the implicit assumption that drivers of risk other than climate change will remain the same, most of the existing studies have failed to account for the influence that socioeconomic development might have on future climate-related health impacts [6].

This crucial issue of temporal scale mismatch was raised more than a decade ago [7,8], the dynamics of vulnerability have been long recognized [9], and several papers have stressed the need for improved understanding of future vulnerability [1,4,10,11,12]. In spite of this, future socioeconomic conditions have been very rarely accounted for until now and projections of human vulnerability are largely lacking [6]. Given that a large share of climate risk assessments serve adaptation purposes, such a practice is likely to introduce systematic bias into climate and health adaptation strategies [3].

Partly to counteract such shortcomings and to foster the use of socioeconomic scenarios and projections within climate risk assessments, the climate change research community has been engaged over the past few years in the development of a new scenario framework, in which climate and socioeconomic scenarios were developed in parallel [13]. This new scenario framework for climate change research comprises a set of greenhouse gas emissions trajectories, namely the Representative Concentration Pathways (RCPs) [14], and a set of global socioeconomic development trends, namely the Shared Socioeconomic Pathways (SSPs) [15]. These global pathways have been designed to be combined in a scenario matrix architecture [16]—assuming that given RCPs can be reached by different SSPs—to explore the wide range of challenges to mitigation and adaptation. While much of the climate research has been focused on understanding the impacts of different RCPs on a wide array of socioeconomic and natural systems [17], very little has been done until now to explore the influence of temporal dynamics of socioeconomic systems—under the SSPs—on future human vulnerability and climate-related health risks [18]. In fact, despite the rapidly growing array of studies making use of both the RCPs and SSPs to explore future climate-related health impacts, the influence of changes in socioeconomic conditions is still largely underestimated and mostly constrained to changes in exposure only, utterly neglecting the effect of changes in human vulnerability [19]. Drawing on this, the aim of this paper is twofold. First, it aims to critically discuss the current state of practice in relation to the use of SSPs in the assessment of future climate-related health impacts, in order to identify and better characterize the research gaps and needs, particularly in terms of availability of socioeconomic projections. Second, through a European case-study, this paper aims to present two innovative methods that complement existing projection methods and have the potential to address the aforementioned research needs.

## 2. Current State of Practice

### 2.1. Shared Socioeconomic Pathways—SSPs

The SSPs are the latest set of IPCC-guided global socioeconomic development trends that provide a global context to guide climate change research at both global and regional levels [15,20]. They are made up of five contrasting global development pathways that depict plausible alternative future states of the society and the environment (see Table S1). They have been purposely designed to span the wide range of socioeconomic challenges to adaptation and mitigation. A substantial body of literature has documented (i) their development [13,15,20,21,22,23,24,25]; (ii) their quantification at the national level up to 2100 for a few key socioeconomic variables—freely available online [26]—such as demography and education [27,28], urbanization [29], economic growth [30,31,32], and land use [33]; (iii) their integration with climate change [34]; and (iv) their links with future atmospheric concentration of greenhouse gases [35,36,37,38].

### 2.2. Extended SSPs

An important feature of the SSPs lies in their flexibility. They have been purposely conceived to be extended, i.e., contextualized, detailed, and eventually quantified for specific regions and/or sectors [39]. Extended versions of the global ‘basic’ SSPs have an increased suitability and usefulness for local and/or sectoral studies, are of greater relevance for policy-making, and are more likely to be used by local stakeholders. A growing number of studies have employed the narratives of the global SSPs to develop extended SSPs for specific regions and/or sectors. So far, extended SSPs include extended SSPs of urban and population development worldwide [40,41], in coastal areas [42,43], and in large cities [44], extended SSPs for health [45,46], for the water sector [47,48], for fisheries [49], for the forestry sector [50], and for food security worldwide [51], in West-Africa [52], and in South-East Asia [53], and extended SSPs for specific regions, e.g., the Barents region [54], the Arctic [55], Tokyo [56], Iberia [57], Scotland [57], the US [58], and Europe [5,59]. 

### 2.3. Integration within Climate-Related Health Impact Assessments

The SSPs have been purposely designed to be used within Impacts, Adaptation, and Vulnerability (IAV) studies. Therefore, they have the potential to enhance the integration of socioeconomic scenarios within future-looking IAV research [39,60] and to improve the comparability between different case studies [61,62]. Over the past few years, a rapidly growing number of IAV studies have made use of the SSPs—coupled with different RCPs—to assess future climate-related health impacts under multiple combinations of socioeconomic and climate scenarios. So far, these studies have been conducted in the fields of food security and hunger risks [53,63,64,65,66,67,68,69], fire risk [70], exposure to vector-borne diseases [71,72], water scarcity [73,74,75,76,77,78], flood risks [79,80,81], air pollution risks [82,83], and heat-related health impacts [84,85,86,87,88,89,90,91,92,93,94,95,96,97].

### 2.4. Research Gaps and Needs

The aforementioned studies that use SSPs and RCPs to explore future climate-related health impacts show a number of recurrent shortcomings, which can be translated into research gaps and needs. These drawbacks are mainly related to the lack of regional and sectoral contextualization of the global SSPs and to the lack of consideration for vulnerability. These are detailed below.

Lack of extended SSPs: a number of existing studies make a straightforward use of the global SSPs’ narratives and of their quantification at the country level, without contextualizing them for the region and/or sector of interest, thus neglecting the processes that are specific to a given region and/or sector and that influence future socioeconomic and environmental trends. The most common practice linked to this shortcoming is to assume a homogeneous population or economic growth rate within an entire country to downscale national projections of the global SSPs at the sub-national scale, without accounting for local socioeconomic processes that may influence the distribution of population or economic growth within the region. For instance, Marsha et al. [92] have applied the global SSPs’ population change rates at the national level (US) to estimate future population growth in Houston, without considering Houston’s specific socioeconomic and urban development. Similarly, Koutroulis et al. [78] and Alfieri et al. [81] have employed national-level quantitative projections of the global SSPs (for GDP and population growth) in their assessments of future water security and flood risk in Europe, without accounting for specific European socioeconomic developments [59]. Another recent example lies in a study [76] in which the authors assumed a similar population growth rate in flood-prone areas as the growth rate at the national level, despite recognizing that population usually tends to grow faster in flood-prone areas than in other places (due to the higher growth rate of low-income populations). In this particular case, such an assumption led to an underestimation of the number of people exposed to river flooding.

Lack of consideration of vulnerability: the overwhelming majority of existing assessments of future climate-related health impacts account only for future exposure (i.e., the future size of the populations exposed to climatic hazards) under different SSPs and neglect the future populations’ vulnerability (i.e., their abilities to prepare for, respond to, and recover from climatic hazards). Such a lack of consideration of vulnerability can be found in most of the existing studies, e.g., in assessment of future heat stress risk [85,88,90,94,95], of future flooding risk [76], of future risk of vector-borne diseases [71], and so on. While most of the aforementioned authors acknowledge that future research should attempt to integrate projections of drivers of human vulnerability, these are still very rarely found. By disregarding the future states of vulnerability, past studies have focused only on the “population effect” (i.e., the influence of population growth on future populations’ exposure) [88,95] and have substantially underestimated the influence that varying levels of socioeconomic development may have on future climate-related health impacts [3]. Up until now, the few studies that have employed projections of vulnerability under the SSPs have been limited to projections of age, education [86], income level, water demand [75,78], urbanization [87], crop demand, and share of livestock [63]. It is also worth noting that most of these projections of drivers of vulnerability have been employed at a very coarse spatial resolution—country-level mainly—, thus limiting their usefulness for policy-making and their intake by local stakeholders as well as their compatibility with climate projections—often realized at 0.5° [98].

To justify such paucity of contextualization of socioeconomic projections under the SSPs and such lack of consideration of future populations’ vulnerability, authors traditionally point out the scarcity of socioeconomic projections, in terms of diversity, spatial scale, consistency with the SSPs, and relevance at local scale (Table 1). This research gap provides a convincing explanation of the aforementioned drawbacks.

This close look at the current state of practice clearly highlights the needs to develop quantitative projections of socioeconomic variables that are consistent with the global SSPs (i.e., linked to global contexts) while accounting for local and sectoral specificities (i.e., making use of extended SSPs), that are produced at relevant spatial and temporal scales—in line with climate models’ outputs and with the scale at which socioeconomic processes happen—, and that cover the broad range of drivers that influence human vulnerability to climate change.

## 3. Methods to Project Drivers of Human Vulnerability

In light of the aforementioned research gaps and needs, I introduce here several methods that have the potential to address the need for quantitative projections of drivers of human vulnerability (e.g., age structure, income, infrastructure, access to resources, urbanization, education, pre-existing medical conditions), consistent with the SSPs, contextualized, and produced at relevant spatial and temporal scales. I first briefly discuss the two main approaches that have been applied so far, namely the use of sectoral models and the spatial disaggregation of existing projections, and then detail two new and innovative methods that can complement the existing approaches. These are based (i) on the matching of existing scenario sets to generate consistent projections and (ii) on the quantification of experts’ opinions coupled with correlation analyses. These have been recently applied to Europe to explore future social vulnerability [5] and future heat-related health impacts [19].

### 3.1. Existing Methods

#### 3.1.1. Use of Sectoral Models

Until now, one of the most common approaches to quantifying the SSPs has been the use of sectoral models. For instance, the global SSPs have been quantified for key socioeconomic variables at the national level with sectoral models such as urbanization models [29], demographic models [27,28], and economic models [31,101]. Worldwide, Jones and O’Neill [40] have employed a gravity model-based approach to produce spatially-explicit projections of population and urbanization patterns. At the regional level, urbanization and demographic sectoral models have been applied, for instance, to produce urbanization and population projections under the SSPs for the 101 largest cities [44], for the coastal zones [42], for the Mediterranean area [43], for Europe [102]. A few other sectoral models have also been used to project—under the SSPs—socioeconomic variables related to water demand and food consumption, mainly at the global scale [47,69,103]. It should be noted here that the use of sectoral models to project drivers of human vulnerability—other than population and urbanization—at the local scale and based on regional/sectoral extensions of the global SSPs has yet to be explored.

#### 3.1.2. Spatial Disaggregation

Another fairly common approach to obtaining projections at a relevant spatial scale in IAV studies is the spatial disaggregation of existing projections, which have been produced at the national scale under the global SSPs [26]. To disaggregate these national-level projections, authors traditionally employed current statistics at sub-national level—considered as the benchmark—and applied country- and SSPs-specific growth/decline rates over all the sub-national units of a given country, ensuring that the relationship between the distance from a given sub-national value to the national mean and the distance from the national mean to the minimum and maximum sub-national values remain similar to those in the benchmark. A few examples of studies that have employed this approach to downscale national projections under the SSPs include (i) Xing et al. [104] who have downscaled projections of population, GDP per capita, and urban population share under SSP2 for 31 provinces in China, (ii) Marsha et al. [92] who have downscaled national projections of GDP and population in the US for each block group of Houston city, and (iii) Rohat et al. [5] who have downscaled projections of education under SSP1, SSP3, and SSP4 at the NUTS-2 level for 30 European countries.

While such a downscaling approach based on current figures is useful to approximate the national projections at a local scale, it fails to account for context-specific characteristics that influence local socioeconomic development trends. To account for these local trends, the downscaling process of the national projections should be informed by an interpretation of the global SSPs’ assumptions at the local scale or by context-specific downscaling scenarios. In Europe, Hurth et al. [105] and Lückenkötter et al. [106] have downscaled national projections of GDP per capita and population density (under the five SSPs) on the basis of the coupling of the latest scenarios of the European Commission Directorate General for Economic and Financial Affairs (ECFIN)—namely the trend and convergence scenarios [107]—with current figures of GDP per capita and population density at very high spatial resolution. The resulting contextualized and downscaled projections of GDP per capita and population density are available at a very high spatial resolution (10 × 10 km spatial grid), accounting for the local context—through the regionalization with the European scenarios—and are consistent with the global SSPs’ national projections. Such projections have been used in Rohat et al. [19] and are expected to be integrated within a number of forthcoming European IAV studies.

### 3.2. Scenario Matching

The use of scenarios in environmental studies has substantially increased over the past decades [108], leading to the development of a large number of different scenario sets in Europe [109,110,111,112]. Although their scientific acceptance and their relevance for climate-related issues may vary [113], these existing scenario sets represent an extremely valuable basis of knowledge regarding the multiple ways the future could unfold and in relation to the impacts of varying levels of socioeconomic development on a range of sectors such as demography, urbanization, housing, economy, health, land use, agriculture, transportation, and so on [114,115]. Although it has been argued that it would be unwise not to employ such a great source of knowledge [116], the re-use of existing scenario sets in IAV studies has been limited until now to the use of their storylines to extend the global SSPs’ narratives [57,58,117]. To my knowledge, the quantitative elements of existing scenario sets (i.e., their quantitative projections) have never been re-used in assessments of future climate-related health impacts. I argue here that the use of existing quantitative projections of previously-developed scenario sets has the potential to address the need for projections of drivers of human vulnerability, provided that they are consistent with the SSPs. The latter consideration is of the utmost importance to ensure the inter-compatibility between SSPs-based IAV studies [39]. The consistency between existing scenario sets and the global SSPs should be rigorously checked, using systematic methods to match the narrative of a given existing scenario with the storyline of a given SSP [50,52,54,58].

Building upon a forthcoming paper [5], I present here the results of a scenario matching approach that was applied to match the global SSPs with three European scenario sets, namely ET2050, DEMIFER, and CLIMSAVE. These can be characterized as follows:

- ET2050 comprises four scenarios of territorial development and cohesion in Europe [118] that have been quantified for variables related to urbanization, accessibility, and transport nodes, at the sub-national level [119]. The four scenarios are named Baseline (Base), MEGAS (A), Regions (B), and Cities (C).
- DEMIFER is made up of five European demographic scenarios [120] that have been quantified for a number of key demographic and lifestyle variables such as labor force, ageing, employment, life expectancy, and different types of migration, at sub-national level [121]. The five scenarios are named Status Quo (STQ), Growing Social Europe (GSE), Expanding Market Europe (EME), Limited Social Europe (LSE), and Challenged Market Europe (CME).
- CLIMSAVE comprises four cross-sectoral European scenarios [122,123,124] that have been quantified for variables related to ecosystems services and provisions and environmental conditions, at high spatial resolution (16 × 16 km) [125]. The four scenarios are named We are the World (WW), Icarus (Ica), Riders on the Storm (RS), and Should I stay or Should I go (SSG).

Employing a systematic scenario matching approach (i.e., a semi-quantitative approach which aims to quantify the similarities between several scenarios originating from different sets of scenarios), authors identified three different groups of scenarios—made of one scenario of each set—each sharing significantly similar storylines (Table 2). These groups of scenarios are then viewed as extended versions of the SSPs (hereafter Ext-SSPs), which showcase an increased relevance (i) at the European level and (ii) for sectors related to human vulnerability, compared to the global SSPs’ storylines.

**Figure 1:**
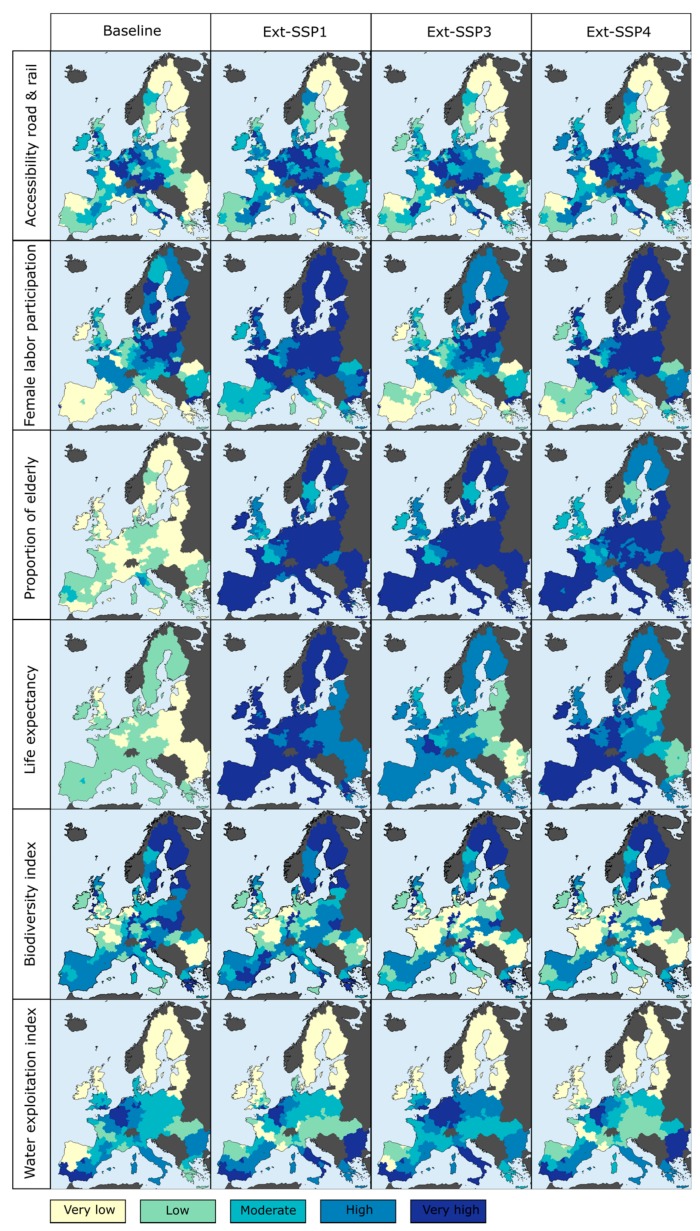
Sample of the available projections of variables related to human vulnerability, under the three extended SSPs (2050) and the baseline (2015) conditions, for the 28 member countries of the European Union, at the NUTS-2 level.

Being made up of a combination of scenarios—one from of each of the four scenario sets—the newly-created Ext-SSPs can be readily quantified through the co-use of the quantitative outputs of each scenario set. As an example, the quantitative projections made under ET2050-B, DEMIFER-GSE, and CLIMSAVE-WW are viewed as consistent with one another and with SSP1—because their respective storylines have been matched—and therefore constitute the quantitative part of Ext-SSP1. In this way, authors were able to readily quantify the three Ext-SSPs at the sub-national level, up to 2050, for a wide number of variables related to territorial development and cohesion (from ET2050 scenarios), demography and lifestyle (from DEMIFER scenarios), and environment (from CLIMSAVE scenarios). A large proportion of these variables are considered as important determinants of human vulnerability and could therefore be integrated within assessments of future climate-related health impacts. Figure 1 and Table 3 present a sample of these readily available and spatially-explicit quantitative projections in Europe under the three Ext-SSPs.

### 3.3. Experts’ Elicitation and Correlation Analyses

For a certain number of determinants of human vulnerability—e.g., those related to health conditions, governance efficiency, or human behavior—quantitative projections under different socioeconomic scenarios simply do not exist (or are extremely scarce) and models are not available or not yet well developed. In such cases, more simplistic approaches may be considered in order to obtain rough projections under the different SSPs. This should be preferred to discarding a variable and/or assuming fixed conditions, particularly if the variable in question is an important driver of vulnerability.

**Figure 2:**
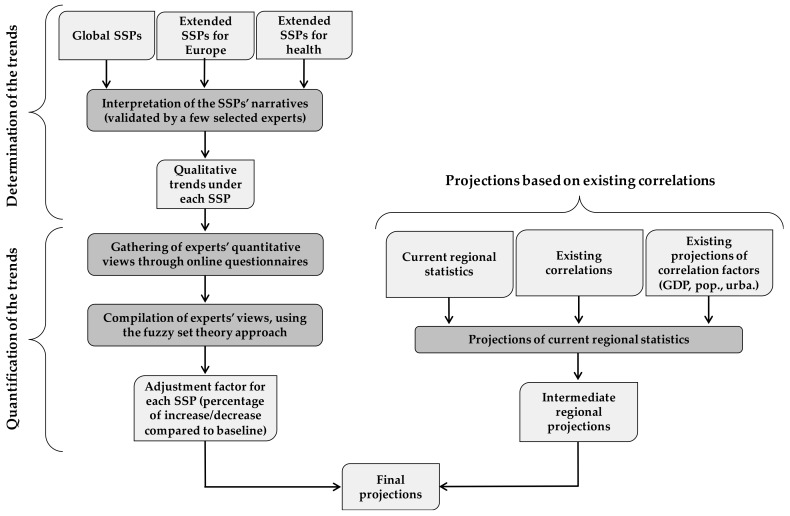
Workflow of the projection method based on experts’ elicitation and correlation analyses.

I present here an innovative approach based on experts’ elicitation and correlation analyses to quantitatively project two significant determinants of vulnerability to heat stress in Europe, namely the proportion of elderly people living alone and the prevalence of overweight. It has been shown that social isolation among the elderly considerably increases the risk of death during extreme temperatures events [126,127,128], mostly due to their lower access to transportation and their lack of support during heat waves [129,130]. Similarly, research has shown that pre-existing medical conditions, such as overweight, lead to significantly higher risk of death during heat waves [126,131,132]. The workflow of this innovative method is presented in Figure 2 and each step is detailed below.

#### 3.3.1. Determination of Local Trends

Due to the global SSPs’ lack of explicitness regarding future developments in public health conditions and in living arrangements of the elderly, I first interpreted the global SSPs—using the existing European SSPs developed within the IMPRESSIONS project [59,117] and the preliminary version of the extended SSPs for health [45]—to determine future trends in the proportion of the elderly living alone and in the prevalence of overweight, under each SSP. The interpretation of these existing extended versions of the SSPs led to a fairly straightforward establishment of future trends in overweight prevalence in Europe, as presented in Table 4. These were validated by three different experts.

Conversely, determining the future trends in the proportion of the elderly living alone was a much less straightforward process, as trends in living arrangements are not mentioned—even implicitly—in the European SSPs’ narratives. Therefore, I first conducted a short literature review to identify the main drivers of living arrangements among the elderly in Europe [133,134,135,136]. With the help of two experts in household composition, I identified the following key drivers: (i) aging of the population, (ii) health conditions (elderly people in better health are more likely to live alone), (iii) economic situation (better-off elderly people are more likely to live alone), (iv) type of society (familistic or individualistic), and (v) social cohesion. In light of these drivers of the elderly’s living arrangements, I then determined the trends direction under each SSP (Table 5). Unlike the trends in future overweight prevalence, the trends in future proportions of elderly people living alone were not only determined for the whole Europe, but also for three different clusters of European countries (namely the Northern cluster, the Central/Western cluster, and the Southern cluster). These clusters were determined based on current figures of the proportion of elderly people living alone, with the current proportion being 40% on average in the Northern cluster, 33% in the Central/Western cluster, and 25% in the Southern cluster. Such elicitation of the trends at the sub-European level allows better accounting for the differential intra-Europe development pathways.

#### 3.3.2. Quantification of the Local Trends Based on Experts’ Elicitation

To quantify the aforementioned trends in the proportion of elderly people living alone and in the prevalence of overweight under each European SSP (EU-SSPs), I employed the fuzzy set theory approach, based on experts’ elicitation [137,138]. In collaboration with a few selected experts, I designed two distinct online questionnaires (Figures S1 and S2) oriented towards health experts and living arrangement experts respectively. In each questionnaire, experts were first presented with a short description of the four EU-SSPs, then with trends in the proportion of elderly people living alone (or in overweight prevalence), under each EU-SSP. Experts were then asked to give their level of agreement with these trends, considering the EU-SSPs’ description given beforehand. They were then asked to give a numerical range, for each scenario trend, of the proportion of elderly people living alone at the sub-European level (or the future overweight prevalence at the European level).

**Figure 3:**
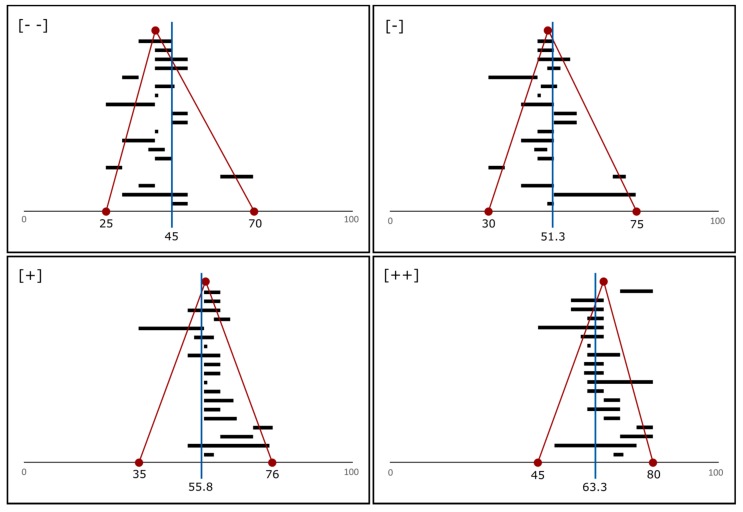
Center of gravity (blue line) for each trend category ([- -] = large decrease; [-] = decrease; [+] = increase; [++] = large increase), computed as the average of the minimum, maximum, and median values (in red) of the experts’ quantitative ranges (in black).

These online questionnaires were distributed to 300 European experts in overweight and 420 European experts in living arrangements, identified through extensive literature research. The response rate approximated 7% for both questionnaires, yielding 21 and 29 different answers for the questionnaires on overweight prevalence and on the proportion of elderly living alone respectively. Based on these experts’ quantitative views, I then determined the center of gravity (using the average of the median, minimum, and maximum values) for each scenario trend [138]. Figure 3 displays such centers of gravity for the prevalence of overweight.

These centers of gravity were then translated into adjustment factors (Table 6), i.e., percentages of increase/decrease (in overweight prevalence at the European level or in the proportion of elderly people living alone at the sub-European level) compared to the baseline (current situation), for the period 2015–2050. These adjustment factors represent the unified experts’ quantitative view on the future trends of these two socioeconomic variables in Europe.

#### 3.3.3. Final Projections

Before producing the final projections of the proportion of elderly people living alone and of the prevalence of overweight, I first computed intermediate regional projections, employing correlation analyses. To do so, I relied on existing correlations between the variable to project and other variables for which projections under the SSPs already exist, e.g., GDP, population, and urbanization. In the case of overweight prevalence, current statistics [139] show that large differences exist across different age groups and urbanization levels. Based on these correlations at the country level and employing existing projections of population (for each age group) and urbanization under the European SSPs—produced at the NUTS-2 level and on a 10 × 10 km spatial grid respectively [106,140] —, I computed intermediate regional projections of overweight prevalence that account for future changes in population structure and urbanization, under each EU-SSPs.

**Figure 4:**
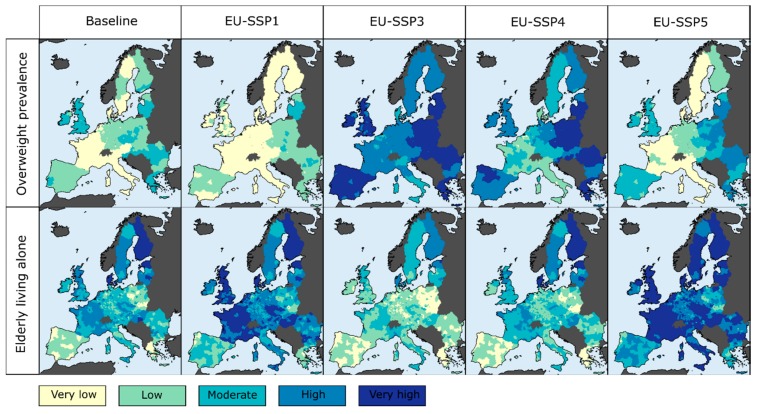
Projections of the prevalence of overweight and of the proportion of elderly living alone, under the four European SSPs (2050) and for current conditions (2015), aggregated at the NUTS-3 level, for the 28 member countries of the European Union.

Employing the scenario-specific adjustment factors determined by the experts, I then computed the final projections of the overweight prevalence under the four EU-SSPs (Figure 4). While these projections are performed at both NUTS-2 level and on a 10 × 10 km spatial grid, the adjustment factors are assumed to be homogeneous over Europe (in the case of the prevalence of overweight) or over each countries’ cluster (in the case of the proportion of elderly people living alone).

It has to be mentioned here that due to the lack of established correlations between the proportion of elderly people living alone and common socioeconomic factors such as GDP, population, and urbanization, no regional intermediate projections of the proportion of elderly people living alone were produced and scenario- and region-specific adjustment factors were directly applied to the current figures at NUTS-3 level

## 4. Discussion

### 4.1. Addressing the Research Needs

The evaluation of the current state of practice (Section 2) clearly highlights the needs to produce quantitative projections of socioeconomic variables that (i) cover the wide range of determinants of human vulnerability to climate change, (ii) are both consistent with the global SSPs and locally-relevant (i.e., based on extended SSPs), and (iii) are available at relevant spatial resolution, in line with climate models’ outputs and with the scale at which socioeconomic processes take place. The two innovative methods presented in this paper—namely the scenario matching approach and the approach based on experts’ elicitation and correlation analyses—showed great potential to address these needs. On the one hand, the scenario matching approach (Section 3.1) led to the quantification of a dozen socioeconomic variables—linked to urbanization, territorial development, demography, employment, and biodiversity—at the sub-national scale (mainly NUTS-2 level), up to 2050, under three different European SSPs. The latter are extended versions of the global SSPs that account for the local specificities of the European Union. Such projections of drivers of human vulnerability have the potential to be integrated in assessments of future climate-related health impacts in Europe, which have so far failed to account for the dynamics of vulnerability [96,141,142].

On the other hand, the approach based on experts’ elicitation and correlation analyses (Section 3.2) showed that singular—but highly important—determinants of human vulnerability, such as social isolation and pre-existing medical conditions, can also be quantitatively projected under the SSPs at relevant spatial and temporal scales. Here I quantified the future proportion of elderly people living alone and the future prevalence of overweight in Europe, at high-spatial resolution (NUTS-3 and 10 × 10 km spatial grid), under four different European SSPs. As for the projections obtained with the scenario matching approach, these projections can be readily included within assessments of future climate-related health risks in Europe, as highlighted in Rohat et al. [19].

In addition to these two innovative approaches, which appear to be useful alternatives, existing methods to quantify the SSPs (e.g., the use of sectoral models and the spatial disaggregation of existing national projections) also show great potential to address the aforementioned research needs. To better address these needs, spatial disaggregation approaches should ideally be informed by local and context-specific downscaling assumptions and/or scenarios. This may enhance the relevance of the outputs for local assessments of climate-related health impacts. Similarly, in order to produce relevant projections for local IAV studies, sectoral models’ inputs should preferably originate from the modelers’ and/or stakeholders’ interpretation of extended versions of the global SSPs rather than from the interpretation of the global SSPs, which largely lack regional and sectoral details [113,138].

### 4.2. Limitations

Although the two innovative approaches presented in this paper have the potential to address the IAV research needs in terms of spatially-explicit, local, and contextualized projections of the wide variety of drivers of human vulnerability—consistent with the SSPs framework—, these are associated with a number of limitations. On the one hand, the scenario matching approach requires the availability of a number of existing scenario sets that showcase specific characteristics, such as detailed narratives that do not contain any assumptions about climate change—so that they can be matched with different RCPs afterwards—and freely available quantitative projections of the socioeconomic variables of interest, performed at relevant temporal and spatial scales. While appropriate scenario sets were easily found at the European level [5], this may not be the case for scenario-poor regions and for studies aiming at matching the SSPs with more local (e.g., national or sub-national) scenario sets. In addition, the scenario matching approach is unlikely to lead to the local/sectoral extension and quantification of all the five SSPs, but rather of a limited number of them. It is indeed very unlikely that existing scenario sets would be found to comprise an analogous scenario for each of the five SSPs. Nevertheless, bearing in mind that most of the IAV studies do not use the five SSPs but rather focus on the few SSPs that best fit with their research needs [84], such a drawback does not appear to limit the potential applicability of the scenario matching approach in assessments of future climate-related health impacts.

On the other hand, the approach based on experts’ elicitation and on correlation analyses makes use of a number of normative judgments and thus provides only rough estimates. For instance, to produce the projections of overweight prevalence with this approach, I assumed that the existing correlations at the national scale—between overweight prevalence and urbanization as well as between overweight prevalence and age groups—were homogeneous within all the sub-national units of a given country and that they will remain the same in the future under all scenarios. Furthermore, I also assumed that the adjustment factors—retrieved from the experts’ quantitative views—were homogeneous over Europe. To account for the potential different regional dynamics across the European countries under each SSP, experts should have been asked to quantify the trends for each of the 28 member countries of the European Union, but this would have inevitably lowered the engagement rate of the experts. In addition to these normative judgments, the projections of overweight prevalence and of the proportion of elderly people living alone could not have been checked for consistency and compared with other projections, as no comparable European scenario exercise was found. Therefore, although they represent the experts’ quantitative views, the accuracy of these projections remains unknown. Finally, although most of the experts showed a high degree of agreement with the trends under each SSPs (Figure S3), their quantitative interpretation of these trends differed substantially (as shown in Figure 3). Employing a similar approach with different experts is likely to yield different results (i.e., different adjustment factors), hence challenging the replicability of such an approach. Further research is needed to explore the uncertainties associated with the use of different groups of experts and to assess the fitness of the fuzzy set theory to combine their quantitative interpretations.

In the same vein, it is also worth mentioning that the use of different methods will inevitably lead to different projections under the same SSPs, posing underappreciated problems of consistency [143] and of inter-comparability across different studies. For instance, population projections in Europe under the SSPs can be chosen from (i) Terama et al. [102], available at the NUTS-2 level, using a regional urbanization growth model and residential preferences under four different European SSPs, (ii) Jones and O’Neill [40], performed with a gravity-based model and available on a 0.125° grid for the five global SSPs, and (iii) Lückenkötter et al. [106], realized with the regional downscaling of national projections, available for the five global SSPs and the two downscaling scenarios, on a 0.1° spatial grid. Although such concern of inter-comparability between different sets of projections is limited to a few common variables (primarily population and GDP), it should be scrutinized and accounted for.

## 5. Conclusions

Following the development of the new scenario framework for climate change research, a rapidly growing number of assessments of future climate-related health impacts are accounting for future socioeconomic conditions, under varying levels of socioeconomic development (i.e., using different SSPs). Nevertheless, as highlighted in this paper throughout the evaluation of the current state of practice, the vast majority of these assessments have focused only on future exposure (i.e., future population patterns) and have failed to account for future populations’ abilities to prepare for, respond to, and recover from climatic hazards. Scrutinizing the research gaps and needs, this paper underlined the rapidly emerging demand for projections of socioeconomic variables that (i) are both consistent with the global SSPs and linked to the local context, i.e., making use of extended SSPs; (ii) are available at relevant spatial and temporal scales; and (iii) cover the broad range of drivers that influence human vulnerability to climate change. So far, such projections are largely lacking. While the well-structured climate modelling community has been engaged in recent decades in the production of high-level climatic projections, the production of socioeconomic projections to inform IAV studies has been left aside [18]. In this paper, I showed that methods to obtain quantitative projections of socioeconomic variables under the SSPs at relevant spatial/temporal scales exist, and that innovative methods can be developed to complement the existing approaches. I presented two innovative approaches—namely the scenario matching approach and an approach based on experts’ elicitation and correlation analyses—that both use contextualized (i.e., extended) European SSPs and that enable the quantification of a wide range of determinants of human vulnerability drivers in Europe, at a relevant spatial (sub-national units) and temporal (2050) scale. Although associated with a number of caveats, these approaches—complemented by existing approaches—show great potential for use by the IAV community to enhance the availability of contextualized projections of drivers of human vulnerability under the SSPs and overcome the supposed scarcity of relevant socioeconomic projections. Assessments of future climate-related health impacts should thus rely on these methods to project and account for future populations’ vulnerability. This way, these studies could explore how socioeconomic changes will affect future health risks under different levels of climate change, e.g., 1.5 °C and 2 °C.

Further research should be conducted to expand the diversity of approaches to produce socioeconomic projections under the SSPs, and to refine the existing projection methods. In particular, further research is needed to (i) better interpret and translate the narratives of the SSPs (both global and extended versions) into quantitative inputs for sectoral models [138,144]—bearing in mind that a given SSP can lead to both negative and positive outcomes on different health issues [46,96], (ii) explore the use of sectoral models developed in other research fields (e.g., housing, energy, and transport planning)—which may provide projections of relevant socioeconomic variables [145], (iii) explore the inter-comparability of the different projection methods, and (iv) explore the potential combinations of existing approaches. Such further research would have the objective of advancing our understanding of future vulnerability patterns, so enabling a more accurate assessment of future climate-related health impacts and the design of more appropriate health adaptation strategies.

## Figures and Tables

**Table 1:** Statements from assessments of future climate-related health impacts based on SSPs and RCPs—with, in addition, one review study (*) and two IAV studies (**) that do not make use of the SSPs but which reflect typical statements found in the literature. These highlights both the need to consider future vulnerability under the SSPs and the lack of available projections to do so.

Study	Statement
[92]	“[…] this study utilized SSP national-level demographic and economic projections rather than city-specific projections of Houston because SSP-based projections were unavailable for the city. The national-level SSP projections […] are likely inaccurate given the city’s rapid growth of racially and ethnically diverse populations.”
[84]	“The health impacts of heat vary by personal susceptibility factors like age, and heat effects might be compounded by concurrent exposures like high air pollution or power outages. Future research could explore [….] whether such characteristics could be projected for future heatwaves with enough resolution to be usefully incorporated into projections.”
[95]	“Our initial exploration of a potentially transformative risk factor for humans only considers population exposure. However, the impacts of heat on humans depend on both exposure and vulnerability, with the latter depending on many other factors including population age, degree and type of pre-existing health conditions, […]. The SSPs may offer a means of exploring potentially critical correlations between heat, population density, vulnerability, and the potential for adaptation.”
[85]	“[…] in this work we only analyzed the change in exposure to extreme heat as a function of a change in the hazard […] and population. To properly estimate a change in risk of mortality/morbidity resulting from this exposure, demographic and socioeconomic characteristics such as age, gender, per capita income and education level should be included into the analysis. However, since projections of these characteristics tend to be relatively coarse and of low confidence, we have not included the demographic and socioeconomic factors in our analysis.”
[97]	“Finally, quantifying exposure is a starting point for estimating future risks, but further work is necessary on vulnerability to the impacts of extreme heat, including population age structure and income, as well as possible changes in social and institutional factors over time, which will play important roles in heat-related impacts.”
[94]	“SSP3 assumes a fragmented world following varied regional social, political, and economic pathways. This may be considered difficult to reconcile with the international collaborative effort that would be required in order to keep the global temperature from exceeding 1.5 °C. However, we consider it here on the grounds that what applies as a general rule globally does not necessarily need to apply for India itself (notwithstanding India’s outsized contribution to world population), and that having a population scenario that spans a larger range will allow a more expanded study of the relation between heatwaves, national population, and *MPEHWd*.”
[91]	“[…] the lethality of deadly climatic conditions can be mediated by various demographic (for example, age structure), socio-economic (for example, air conditioning, early warning systems) and urban planning (for example, vegetation, high albedo surface) factors that were not considered in our study. Consideration of these factors would improve the understanding of global human vulnerability to heat exposure […].”
[71]	“Other study limitations are related to human and mosquito behavior. […] how human interventions aimed at reducing *Ae. Aegypti* populations may change in the future is unknown. For example, controversial releases of genetically-modified ‘sterile’ male mosquitoes may become more common in the future, and, if they do, would differ between the SSPs. Additionally, how other human factors such as cultural practices, water access, urbanization, transportation networks and global trade may evolve and impact the spread of *Ae. aegypti* is unclear.”
[76]	“[…] the SSP characterizations are preliminary. […] only simple indicators of changes in exposure to water resources scarcity and river flood frequency are used. These indicators consider only population, and do not incorporate other differences between socio-economic scenarios such as differences in water withdrawals or rate of urbanization. Including such additional dimensions would increase the differences between the SSPs. Future assessments should include more sophisticated measures of exposure and impact […].”
[73]	“In future studies, we would like to account for more demographic characteristics in addition to growth, i.e., age, sex, education, and income, which are likely to be stronger factors for demographic change in the 1.5 ºC target. However, we currently lack the required sophisticated data.”
[75]	“[…] we used a simplistic model to estimate industrial and municipal water use. Progress in this area of modeling has long been obstructed by a lack of data, but further efforts are needed. […] the water use scenario that is used significantly affects the results; hence further efforts are needed to establish consistent scenarios.”
[74]	“To come to a full risk assessment framework more work needs to be done to make the transfer from risk estimates in terms of exposed population towards estimates covering ‘economic’ impacts. A first step therein should be to include vulnerability, including: the sensitivity of a population to water scarcity, the available infrastructure and (financial) resources to cope with water scarcity, […] and capability of the responsible government to deal with water scarcity in a quick and efficient manner.”
[72] *	“[…] final suggestion related to making better use of the new generation of socioeconomic scenarios. It is somewhat ironic that climatic impacts, adaptation and vulnerability (IAV) research, which is so dependent upon assumptions about socioeconomic development, has tended to underutilize socioeconomic scenarios. This is no different for the health sector, but there are opportunities to rectify the situation. […] one solution would be for climate change and health researchers to work to extend the SSPs so that they have more specific health-related variables. […] one key issue is the availability and parameterization of relevant vulnerability indicators within the SSPs. […] the availability of high-resolution projections for broader-level vulnerable indicators such as income distribution, population, health, and governance would be an important starting point.”
[99] **	“[…] it was decided to base adaptive capacity on present day data rather than future projections because it is much harder to obtain future projections of relevant socioeconomic data than it is for climate data: the great uncertainty inherent in any socioeconomic projections would contribute to the multiplication of overall model uncertainties.”
[100] **	“Although vulnerability is dynamic and changes over time, there is no quantitative information available about how this may affect damages. Hence, we assumed no future changes in vulnerability.”

**Table 2:** Groups of scenarios sharing similar storylines, matched with the scenario matching approach [5]. Each group constitutes a given extended SSP (Ext-SSP).

Group of Scenarios	Global SSPs	ET2050 Scenarios	DEMIFER Scenarios	CLIMSAVE Scenarios
Ext-SSP1	SSP1	B	GSE	WW
Ext-SSP3	SSP3	Base	CME	Ica
Ext-SSP4	SSP4	A	EME	RS

**Table 3:** Quantitative projections of relevant variables related to human vulnerability that are readily available through the scenario matching approach for the three Ext-SSPs. All these projections cover the 28 member countries of the European Union.

Variable	Spatial and Temporal Scales	Source
Population per sex and age group	NUTS-2, 2015–2050, 10-year steps	DEMIFER
Proportion of elderly and young		
Dependency ratios (economic and old age)		
Labor force participation per sex and age group		
Migration rates per type (international, inter-country, and extra-Europe)		
Life expectancy per sex		
Urbanization	NUTS-3, 1990–2050, yearly	ET2050
Accessibility per type (road, rail, air, freight)		
Investment in transportation networks		
Transportation network improvements		
Water use (water exploitation index, manufacturing water withdrawal, irrigation usage, total water use)	~16 × 16 km, 2020, 2050	CLIMSAVE
Biodiversity (Shannon index)		
Agriculture (productivity, type of crops, intensity)		

**Table 4:** Trends in future prevalence of overweight in Europe under each European SSP (EU-SSPs), based on the interpretation of the existing EU-SSPs [59] and extended SSPs for health (the latest version of the extended SSP for health [46] were not yet available when this research was conducted, so the preliminary version [45] was used instead).

EU-SSPs	Citations Extracted from the Narratives of the European SSPs and the Health-SSPs	Trend in Prevalence of Overweight in Europe
EU-SSP1	“Population health improves significantly” “Increased emphasis on enhancing health and health care functions” “Reduced burden of health outcomes” “Changes in dietary patterns to lower burden of some chronic diseases” “High investments in human health and education”	Large decrease
EU-SSP3	“Population health decreases significantly” “Countries experience double burden of infectious and chronic climate-related health outcomes” “Reduced funding for surveillance and monitoring programs” “Low investments in human health and education” “Phasing out of social security system”	Large increase
EU-SSP4	“Unequal world, with limited access to high quality education and health services” “Lower burden of some chronic diseases from changes in dietary patterns” “High investments in human health and education for elites only, low for others”	Increase
EU-SSP5	“World attains human sustainable goals” “Health improves significantly, but not as much as in SSP1” “Because the challenges for local management of environmental quality are larger, the burden of chronic diseases is somewhat higher than in SSP1” “High investments in human health and education”	Decrease

**Table 5:** Trends in future proportion of elderly living alone in Europe under each European SSP (EU-SSPs), at the European level (EU) and for each of the three countries’ clusters.

EU-SSPs	EU	Northern	Central/Western	Southern
EU-SSP1	Increase	Stable	Increase	Increase
EU-SSP3	Large decrease	Decrease	Large decrease	Decrease
EU-SSP4	Decrease	Stable	Decrease	Decrease
EU-SSP5	Large increase	Increase	Large increase	Large increase

**Table 6:** Scenario-specific adjustments factors, i.e., percentage of increase or decrease for the period 2015–2050, for overweight prevalence at the European level and for the proportion of elderly living alone at the Sub-European level.

Variable	Area	Trend	Center of Gravity	Adjustment Factor (%)
Overweight prevalence	Europe	Large increase	63.3	+19.5
		Increase	55.8	+0.1
		Decrease	51.3	−14.1
		Large decrease	45	−27.6
Proportion of elderly living alone	Northern Europe	Increase	46.6	+16.7
		Decrease	33.6	−15.8
	Central/Western Europe	Large increase	42.7	+29.3
		Increase	39.5	+19.7
		Decrease	30.8	−6.5
		Large decrease	26.7	−19.2
	Southern Europe	Large increase	34.5	+38.0
		Increase	29.3	+17.3
		Decrease	23.7	−5.3

## Supplementary Materials

The following are available online at http://www.mdpi.com/1660-4601/15/3/554/s1, Table S1: Short narratives of the five Shared Socioeconomic Pathways; Figure S1: Questionnaire distributed to experts in overweight; Figure S2: Questionnaire distributed to experts in household composition; Figure S3: Experts’ level of agreement with the presented trends.
