# Supplementary material for: Projecting Drivers of Human Vulnerability under the Shared Socioeconomic Pathways
Source: Int J Environ Res Public Health. 2018 Mar 19;15(3):554. doi: 10.3390/ijerph15030554 (PMC5877099; doi:10.3390/ijerph15030554)
Supplement: Supplementary file 1 [file ijerph-15-00554-s001.pdf]

International Journal of Environmental Research and Public Health

***** Supplementary material *****

Projecting drivers of human vulnerability under the Shared Socioeconomic Pathways

Guillaume Rohat [1, 2]

[1]Institute for Environmental Sciences, University of Geneva, Switzerland
[2]Faculty of Geo-Information Science and Earth Observation, University of Twente, The Netherlands

Table S1 – Short narratives of the five Shared Socioeconomic Pathways [1,2]

**SSP1** Sustainability – Taking the green road	*"The world shifts gradually, but dramatically, toward a more sustainable path. Management of the global commons slowly improves, educational and health investments accelerate the demographic transition, and concerns with economic growth shift toward the implications for human well-being. Overall energy and resource use is reduced over the longer term, and renewables become more attractive."*
**SSP2** Middle of the road	*"Development and income growth proceeds unevenly, with only some countries making relatively good progress. Global and national institutions work toward but make slow progress in achieving sustainable development goals. Technological developments proceed apace, but without fundamental breakthroughs. Global population growth is moderate; education investments are not high enough to accelerate the transition to low fertility rates in low-income countries."*
**SSP3** Regional rivalry – A rocky road	*"Growing interest in regional identity and concerns about competitiveness and security push countries to increasingly focus on domestic or, at most, regional issues. Policies are oriented toward security, and countries focus on achieving energy and food security goals within their own region, at the expense of broader-based development. Population growth is low in industrialized and high in developing countries. Inequities are high, especially in developing countries. There is growing resource intensity and fossil fuel dependency along with difficulty in achieving international cooperation."*
**SSP4** Inequality – A road divided	*"In this world, inequalities increase, both between and within countries. Vulnerable groups are largely outside the mainstream globalized economic system and have little representation in national and global institutions, which emphasize international competitiveness. This is a world with low social cohesion, and regularly in social conflict and unrest. Power becomes more concentrated in a relatively small political and business elite, even in democratic societies. Energy companies diversify their energy sources to hedge against price fluctuations, investing in carbon-intensive fuels such as coal and unconventional oil, but also low-carbon energy sources."*
**SSP5** Fossil-fueled development – Taking the highway	*"Driven by the economic success of industrialized and emerging economies, this world places increasing faith in competitive markets, innovation, and participatory societies to produce rapid technological progress and development of human capital as the path to sustainable development. Global markets are increasingly integrated, and there are strong investments in health, education, and institutions to enhance human and social capital. At the same time, the push for economic and social development is coupled with the exploitation of abundant fossil fuel resources and the adoption of resource and energy intensive lifestyle around the world."*

Figure S1 – Online questionnaire distributed to the experts in overweight and obesity

Questionnaire
Future proportion of overweight people (adults) in Europe: experts-based quantification

Short description of the four European socioeconomic scenarios' characteristics

	**Socioeconomic scenarios**			
**Determinants**	**A - Sustainability**	**B - Conventional development**	**C - Inequality**	**D - Fragmentation**
Population health conditions	Large improvement	Improvement	Improvement for elites, decrease for other	Large decrease
Environmental conditions	Large improvement	Improvement	Inequal	Large decrease
Human health investments	High	High	High for elites only	Low
Education investments	High	High	High for elites only	Low
Social capital and cohesion	High	High	Low	Low
Changes of dietary patterns	Major changes to lower burden of some chronic diseases	No change	A few changes in elites to lower burden of some chronic diseases	No change
Governance	High collaboration, efficient, coordinated efforts to achieve SDGs	High collaboration, focusing on business and economic development	Weak institutions, governments focusing on national security and internal issues	Weak governance and institutions, lack of collaboration and consensus
Ageing of the population	High	Medium	High	Medium

Trends of proportion of overweight people (adults only, BMI >=25) in Europe for the four socioeconomic scenarios:

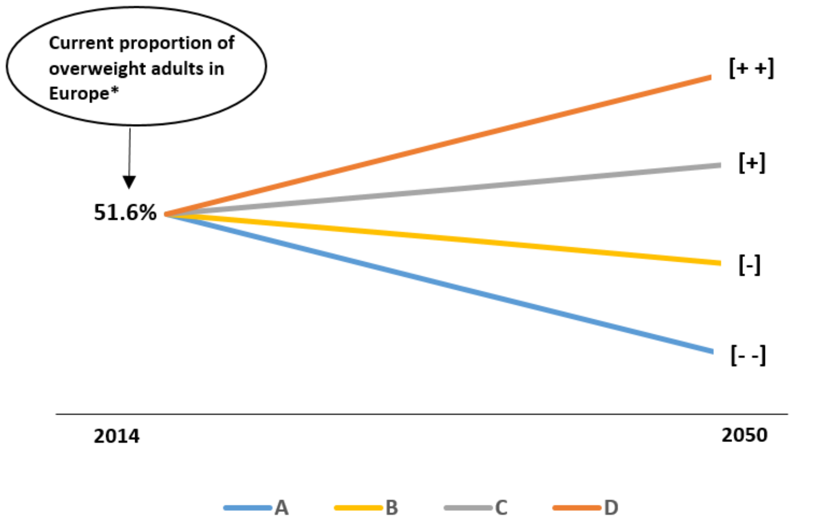

*source: Eurostat

<u>Question 1</u>: In view of the scenarios' characteristics listed in the table above, to what degree do you agree with the trends of proportion of overweight people (adults) in Europe presented in the figure above?

- 1 – Strongly disagree
- 2
- 3
- 4
- 5 – Strongly agree

<u>Question 2</u>: Please provide numerical ranges of the future proportion (in %) of overweight people (adults) in Europe in 2050, for each trend category (e.g. [++]: 59-63%). Overlapping between ranges is possible but not compulsory.

- [++] ____________________________
- [+] ____________________________
- [-] ____________________________
- [- -] ____________________________

Figure S2 – Online questionnaire distributed to the experts in household composition

Questionnaire
Future proportion of elderly (>65 years) living alone in Europe: experts-based quantification

Short description of the four European socioeconomic scenarios' characteristics

	**Socioeconomic scenarios**			
**Determinants**	**A - Sustainability**	**B - Fragmentation**	**C - Inequality**	**D - Conventional development**
**Social cohesion**	High and familistic	Moderate	Low	High but individualistic
**Population health conditions**	Large improvement	Large decrease	Improvement for elites only	Improvement
**Economic conditions**	Large improvement	Large decrease	Improvement for elites only	Large improvement
**Ageing of the population**	High	Medium	High	Medium
**Governance efficiency and orientation**	Efficient, towards quality of life and human capital	Very weak, no particular orientation	Weak, towards national security	Efficient, towards business and economic development
**Investment in health and social support**	Very high	Low	High for elite only	High

<u>*Question 1*</u>*: What is your geographical region of expertise, if any?*

- ○ Northern Europe (*redirected to A*)
- ○ Central / Western Europe (*redirected to B*)
- ○ Southern Europe (*redirected to C*)
- ○ All Europe (*redirected to ABC*)

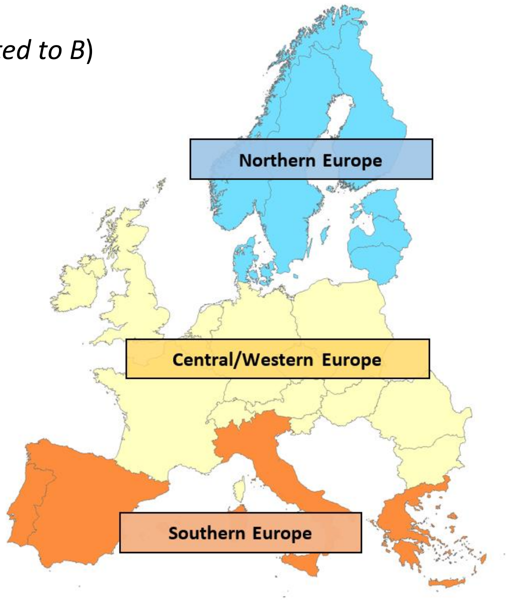

A. Northern Europe

Trends of proportion of elderly (>65 year) living alone in Northern Europe for the four socioeconomic scenarios:

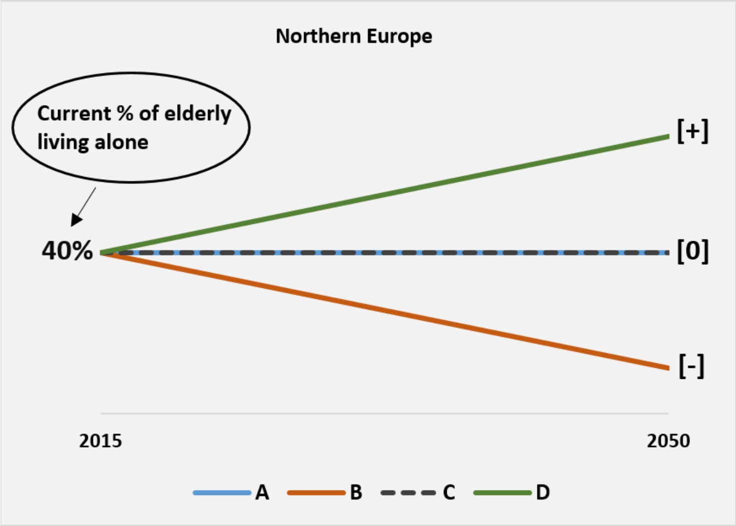

<u>Question A.1</u>: In view of the scenarios' characteristics listed in the table above, to what degree do you agree with the trends of elderly living alone in Northern Europe presented in the figure above?

- 1 – Strongly disagree
- 2
- 3
- 4
- 5 – Strongly agree

<u>Question A.2</u>: Please provide numerical ranges of the future proportion (in %) of elderly living alone in Northern Europe in 2050, for each trend category (e.g. [+]: 47-50%). Overlapping between ranges is possible but not compulsory.

- [+] ______________________________
- [-] ______________________________

B. Central/Western Europe

Trends of proportion of elderly (>65 year) living alone in Central/Western Europe for the four socioeconomic scenarios:

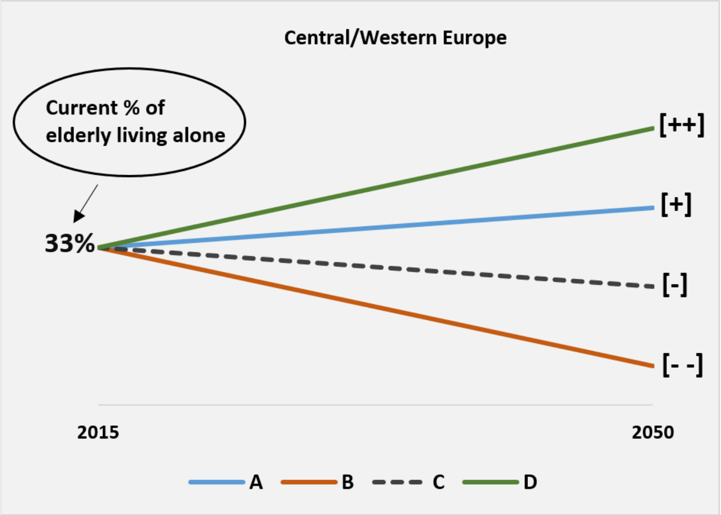

<u>Question B.1</u>: In view of the scenarios' characteristics listed in the table above, to what degree do you agree with the trends of elderly living alone in Central/Western Europe presented in the figure above?

- 1 – Strongly disagree
- 2
- 3
- 4
- 5 – Strongly agree

<u>Question B.2</u>: Please provide numerical ranges of the future proportion (in %) of elderly living alone in Central/Western Europe in 2050, for each trend category (e.g. [++]: 41-45%). Overlapping between ranges is possible but not compulsory.

- [++] ______________________________
- [+] ______________________________
- [-] ______________________________
- [- -] ______________________________

C. Southern Europe

Trends of proportion of elderly (>65 year) living alone in Southern Europe for the four socioeconomic scenarios:

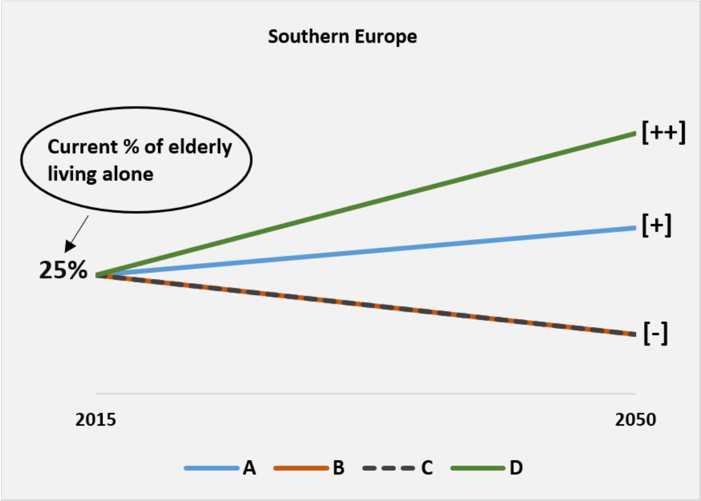

<u>Question C.1</u>: In view of the scenarios' characteristics listed in the table above, to what degree do you agree with the trends of elderly living alone in Southern Europe presented in the figure above?

- 1 – Strongly disagree
- 2
- 3
- 4
- 5 – Strongly agree

<u>Question C.2</u>: Please provide numerical ranges of the future proportion (in %) of elderly living alone in Southern Europe in 2050, for each trend category (e.g. [++]: 32-35%). Overlapping between ranges is possible but not compulsory.

- [++] ______________________________
- [+] ______________________________
- [-] ______________________________

Figure S3 – Experts' level of agreement with the presented trends (1= strongly disagree; 5= strongly agree)

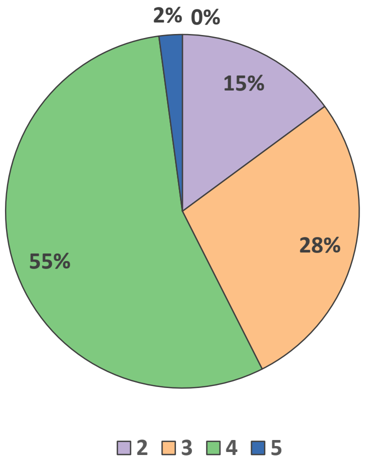

References

1. O'Neill, B.C. *et al.* A new scenario framework for climate change research: The concept of shared socioeconomic pathways. *Climatic Change* **2013**, *122*, 387-400.
2. O'Neill, B.C. *et al.* The roads ahead: Narratives for shared socioeconomic pathways describing world futures in the 21st century. *Glob. Environ. Chang.* **2017**, *42*, 169-180.
